# The recent emergence in hospitals of multidrug-resistant community-associated sequence type 1 and *spa* type t127 methicillin-resistant *Staphylococcus aureus* investigated by whole-genome sequencing: Implications for screening

**DOI:** 10.1371/journal.pone.0175542

**Published:** 2017-04-11

**Authors:** Megan R. Earls, Peter M. Kinnevey, Gráinne I. Brennan, Alexandros Lazaris, Mairead Skally, Brian O’Connell, Hilary Humphreys, Anna C. Shore, David C. Coleman

**Affiliations:** 1 Microbiology Research Unit, Dublin Dental University Hospital, University of Dublin, Trinity College, Dublin, Ireland; 2 National MRSA Reference Laboratory, St. James’s Hospital, Dublin 8, Ireland; 3 Department of Microbiology, Beaumont Hospital, Dublin, Ireland; 4 Department of Clinical Microbiology, School of Medicine, Trinity College Dublin, St. James’s Hospital, Dublin 8, Ireland; 5 Department of Clinical Microbiology, Royal College of Surgeons in Ireland Education and Research Centre, Beaumont Hospital, Dublin, Ireland; Rockefeller University, UNITED STATES

## Abstract

Community-associated *spa* type t127/t922 methicillin-resistant *Staphylococcus aureus* (MRSA) prevalence increased from 1%-7% in Ireland between 2010–2015. This study tracked the spread of 89 such isolates from June 2013-June 2016. These included 78 healthcare-associated and 11 community associated-MRSA isolates from a prolonged hospital outbreak (H1) (*n* = 46), 16 other hospitals (*n* = 28), four other healthcare facilities (*n* = 4) and community-associated sources (*n* = 11). Isolates underwent antimicrobial susceptibility testing, DNA microarray profiling and whole-genome sequencing. Minimum spanning trees were generated following core-genome multilocus sequence typing and pairwise single nucleotide variation (SNV) analysis was performed. All isolates were sequence type 1 MRSA staphylococcal cassette chromosome *mec* type IV (ST1-MRSA-IV) and 76/89 were multidrug-resistant. Fifty isolates, including 40/46 from H1, were high-level mupirocin-resistant, carrying a conjugative 39 kb *iles2*-encoding plasmid. Two closely related ST1-MRSA-IV strains (I and II) and multiple sporadic strains were identified. Strain I isolates (57/89), including 43/46 H1 and all high-level mupirocin-resistant isolates, exhibited ≤80 SNVs. Two strain I isolates from separate H1 healthcare workers differed from other H1/strain I isolates by 7–47 and 12–53 SNVs, respectively, indicating healthcare worker involvement in this outbreak. Strain II isolates (19/89), including the remaining H1 isolates, exhibited ≤127 SNVs. For each strain, the pairwise SNVs exhibited by healthcare-associated and community-associated isolates indicated recent transmission of ST1-MRSA-IV within and between multiple hospitals, healthcare facilities and communities in Ireland. Given the interchange between healthcare-associated and community-associated isolates in hospitals, the risk factors that inform screening for MRSA require revision.

## Introduction

Staphylococcus aureus can cause a wide variety of diseases ranging in severity from superficial skin infections to life-threatening invasive infections such as necrotizing pneumonia, endocarditis and sepsis [1, 2]. Methicillin-susceptible *S*. *aureus* become methicillin-resistant *S*. *aureus* (MRSA) upon acquisition of the Staphylococcal Chromosomal Cassette *mec* (SCC*mec*) mobile genetic element. SCC*mec* harbors *mecA* or *mecC*, both of which encode alternate penicillin-binding proteins, which mediate resistance to almost all β-lactam antibiotics [3–5]. The *mecA* gene encodes the penicillin-binding protein known as PBP2a, whereas *mecC* encodes a homolog that shares 62% amino acid identity with MecA proteins previously described in MRSA [6, 7]. MRSA constitute a major burden in healthcare and community settings worldwide.

Accurate characterization and tracking of nosocomial MRSA strains is essential to reduce the spread of infection. Previously, sequence-based typing approaches for MRSA focused on molecular typing methods that characterize small sections of the genome including multilocus sequence typing (MLST), SCC*mec* and *spa* typing [8, 9]. However, in recent years whole-genome sequencing (WGS) has revolutionized tracking the spread of MRSA in both outbreak and long-term epidemiological investigations [10, 11]. Analysis of single nucleotide variations (SNVs) between isolates provides higher-level discrimination compared to traditional molecular typing techniques, although data analysis involves complex bioinformatics [10, 12–14]. Data from *S*. *aureus* sequence types (STs) ST22, ST2257, ST30 and ST36 showed that multiple colonies recovered from a single patient swab can vary by up ≤40 SNVs [15]. This 40 SNV intra-host strain variation threshold has since been used to infer relatedness between isolates and to identify transmission events [10, 16]. In addition to SNV analysis, whole-genome MLST (wgMLST), involving >1,800 genome-wide loci, has been applied to investigate relationships between MRSA isolates [17]. This approach currently provides the optimal resolution to infer phylogenetic relatedness among isolates, permitting the identification of possible, probable, or unlikely cases of epidemiological linkage. Core-genome MLST (cgMLST), which excludes accessory genome loci, is a refinement of wgMLST based on genes present in each isolate genome [18].

MRSA are largely categorized as healthcare-associated (HCA) and community-associated (CA). While HCA-MRSA often exhibit resistance to multiple antimicrobial agents and typically infect individuals who are immunocompromised or have specific risk factors, CA-MRSA have traditionally been associated with colonization/infection of healthy individuals and susceptibility to most antibiotics [19]. In recent years however, distinctions between these groups have become blurred. CA-MRSA clones have become prevalent in some nosocomial settings [20, 21] and multidrug-resistant (MDR) CA-MRSA are being increasingly reported [22, 23]. Furthermore, genetic markers including SCC*mec* IV and V or virulence determinants such as the Panton-Valentine leukocidin (PVL) toxin, previously considered to be exclusively associated with CA-MRSA, are no longer reliable indicators [22, 24–26].

Since its emergence in the 1990s as the first CA-MRSA clone [27], ST1-MRSA-IV has arisen in diverse settings. Following its initial success as a CA clone [19, 28], ST1-MRSA-IV has been associated with HCA-colonization and infection in North and South America, Europe, the Middle East and Asia [21, 29–31]. More recently, ST1-MRSA-IV *spa* type (t) 127 has been recovered from companion animals, livestock and livestock produce in Italy, Austria and Hungary [32–36].

MRSA has been endemic for four decades in Irish hospitals, since first reported in 1971 [10, 24, 37–43]. While predominant MRSA clone replacement has occurred several times in Ireland [40], ST22-MRSA-IV has been the predominant nosocomial clone since 2002 [10, 44]. Characterization of sporadically-occurring MRSA in Ireland between 2000 and 2012 identified an extensive range of MRSA genotypes and the emergence of several PVL-negative CA-MRSA clones, including ST1-MRSA-IV-t127, which accounted for just 2.3% (2/88) of isolates [40]. In 2010, <1% of all isolates identified at the Irish National MRSA Reference Laboratory (NMRSARL) were t127, or the closely related t922, while in 2015, these isolates accounted for 7% of all those detected [45, 46].

This study comprehensively characterized 89 MRSA-t127/t922, isolates, recovered between 2013–2016 from multiple hospital, healthcare and community sources in Ireland, including a protracted hospital outbreak, in order to investigate isolate relationships and the extent of their spread. Core-genome MLST and SNV analyses revealed the recent emergence and extensive spread of two closely related strains and multiple sporadic strains of ST1-MRSA-IV-t127/t922. Isolates of this clone were predominantly MDR and frequently high-level mupirocin resistant (Hi-MupR), the latter of which can negatively affect efforts to eradicate carriage in colonized individuals.

## Results

### MRSA isolates

Eighty-seven t127 and two t922-MRSA isolates identified by the NMRSARL from June 2013-June 2016 were investigated. The majority of isolates (78/89; 87.6%) were HCA-MRSA, 46/78 (59.0%) of which were recovered from infections or colonization screening during a protracted outbreak in a single hospital (H1) from November 2013-February 2016. The remaining HCA-MRSA isolates (32/78; 41.0%) were recovered in 16 separate Irish hospitals (H2-H17) and four other healthcare facilities (HCFs). Eleven isolates (11/89, 12.4%) were CA-MRSA. Four isolates from hospitals/HCFs other than H1 and one CA-MRSA isolate were recovered from patients with recent hospital H1 admission history (see S1 Table for details). The majority of isolates (75/89; 84.3%) were MDR, exhibiting phenotypic resistance to three or more clinically relevant antibiotic classes in addition to β-lactams, including aminoglycosides, macrolides, mupirocin, tetracycline and fusidic acid (Table 1). DNA microarray profiling confirmed the presence of corresponding resistance genes including *aphA3* (79/89 isolates; 88.8%) and *aadD* (1/89; 1.1%) encoding aminoglycoside resistance, *erm*(C) (71/89; 79.8%) encoding macrolide resistance, *mupA (ileS2)* (52/89; 58.4%) encoding high-level mupirocin resistance, *tet*(K) (39/89; 43.8%) and *tet*(M) (1/89; 1.1%) encoding tetracycline resistance and *fusB* (2/89; 2.2%) and *fusC* (7/89; 7.9%) encoding fusidic acid resistance. The majority of isolates (55/89; 61.8%) harbored at least one *qac* gene encoding resistance to quaternary ammonium compounds. DNA microarray profiling also confirmed that all isolates belonged to clonal complex (CC) 1, harbored SCC*mec* IV (CC1-MRSA-IV) and carried the enterotoxin gene *seh*, which is typically associated with CC1-MRSA. The immune evasion cluster (IEC) genes *sak* and *scn* (IEC type E) were detected in 88/89 (98.9%) isolates. Isolate details are shown in S1 Table.

**Table 1:** Phenotypic resistance patterns of 89 ST1-MRSA-IV t127/t922 MRSA isolates investigated to six clinically relevant antibiotic classes.

Isolate source (no. of isolates)	No. of isolates exhibiting resistance to antibiotic classes (%)^a^						
	BL	AG	ML	MUP	TET	FUS	MDR^b^
**Healthcare-associated (78)**	78 (100)	70 (89.7)	72 (92.3)	46 (59.0)	67 (85.9)	10 (12.8)	66 (84.6)
Hospital 1 (46)	46 (100)	41 (89.1)	41 (89.1)	40 (87.0)	38 (82.6)	1 (2.2)	38 (82.6)
Hospitals 2–17 and four other HCFs (32)	32 (100)	29 (90.6)	31 (96.9)	6 (18.8)	29 (90.6)	9 (28.1)	28 (87.5)
**Community-associated (11)**	11 (100)	9 (81.8)	9 (81.8)	4 (36.4)	8 (72.7)	1 (9.1)	9 (81.8)
**Total (89)**	89 (100)	79 (88.8)	81 (91.0)	50 (56.2)	75 (84.3)	11 (12.4)	75 (84.3)

^a^In each row, all percentages are expressed as a proportion of the total number of isolates investigated.

^b^Multidrug-resistant (MDR) isolates were defined as those exhibiting phenotypic resistance to three or more classes of clinically relevant antibiotics in addition to β-lactams.

Abbreviations: AG, aminoglycoside antibiotics; BL, β-lactams; FUS, fusidic acid; HCFs, healthcare facilities; ML, macrolide/lincosamides; MUP, mupirocin; TET, tetracycline.

### CC1-MRSA-IV isolate relatedness based on WGS

**Fig 1:**
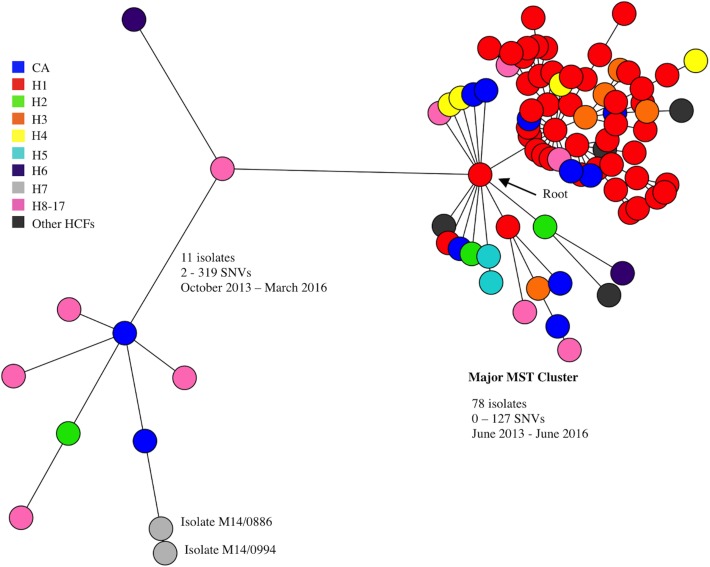
A minimum spanning tree based on core-genome multilocus sequence typing data from 89 ST1-MRSA-IV/t127 or t922 isolates. The pairwise single nucleotide variation (SNV) range between isolates inside and outside of the major minimum spanning tree (MST) cluster and their recovery time frame are indicated. Two outlier isolates, M14/0994 and M14/0886, were recovered 49 days apart from different patients on separate wards in hospital H7 and differed from each other by only two SNVs. The locations from which the isolates were recovered are indicated in the color legend. One isolate was recovered from each of hospitals H8-H17. Abbreviations: CA, community associated; H, hospital; HCFs, other healthcare facilities.

All 89 isolates were assigned to ST1. Based on cgMLST, the majority of isolates (78/89; 87.6%) grouped within one major minimum spanning tree (MST) cluster, while outlier isolates (11/89; 12.4%) dispersed throughout the remainder of the MST (Fig 1). The 78 major MST cluster isolates, both HCA (68/78; 87.2%) and CA (10/78; 12.8%), were recovered over three years and differed from each other by 0–127 SNVs. *In vivo* SNV analysis revealed that two sets of 13 colonies, each isolated from a single patient swab (the patients from which study isolates M13/0653 and M15/0221 were recovered, respectively), differed by 0–36 SNVs and 0–43 SNVs, respectively. Based on this maximum intra-strain difference of 43 SNVs, isolates within the major MST cluster, recovered over three-years and differing by ≤127 SNVs, were deemed closely related. The majority of MDR isolates were located in the major MST cluster (74/75, 98.6%).

**Fig 2:**
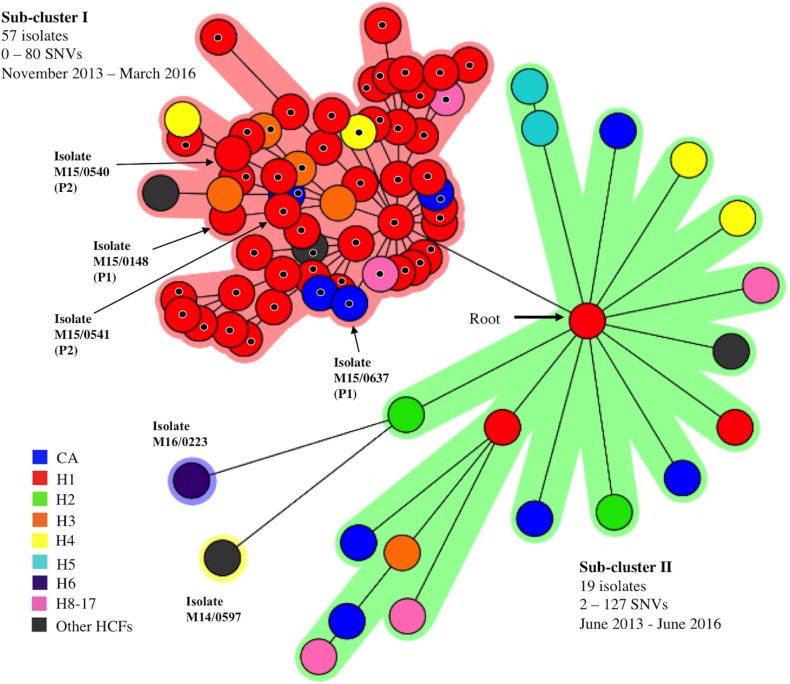
A minimum spanning tree based on core-genome multilocus sequence typing data from 78 ST1-MRSA-IV/t127 or t922 isolates within the major cluster identified in Fig 1. Two sub-clusters were evident within the major cluster: sub-cluster I; highlighted in pink and sub-cluster II; highlighted in green. The pairwise single nucleotide variation (SNV) range between isolates within each sub-cluster and their recovery time frame are indicated. Two intra-cluster outliers were present (M16/0223 and M14/0597), highlighted in purple and yellow, respectively. Two isolate pairs, each recovered from separate patients (patient P1, M15/0148; *iles2*-negative/mupirocin-susceptible and M15/0637; *iles2*-positive/high-level mupirocin-resistant) and patient P2 (M15/0540; *iles2*-positive/mupirocin-susceptible and M15/0541; *iles2*-positive/high-level mupirocin-resistant) are indicated using arrows. M15/0540 harbored an *iles2*-encoding plasmid with a premature stop codon within the *iles2* gene. The locations from which the isolates were recovered are indicated in the color legend. One isolate was recovered from each of hospitals H8-H17. Isolates that exhibited high-level mupirocin resistance are indicated using a small black circle overlaying the relevant isolate node. Abbreviations: CA, community associated; H, hospital; HCFs, other healthcare facilities.

Two sub-clusters and two “intra-cluster outliers” were identified within the major MST cluster following the generation of a second MST based on cgMLST loci from isolates within the major MST cluster only (Fig 2). Isolates within each sub-cluster differed from each other at ≤58 cgMLST loci. Isolates within sub-clusters I and II differed by 0–80 and 2–127 SNVs, respectively and included all 46 H1 outbreak isolates (Fig 2). All isolates in sub-cluster I (57/57) and 89.5% (17/19) of those in sub-cluster II were MDR.

Sub-cluster I (Fig 2) consisted of 57 isolates recovered over 28-months (November 2013–March 2016), including the majority of H1 outbreak isolates (43/46; 93.5%) and isolates recovered from hospitals H3 (*n* = 4), H4 (*n* = 2), H13 (*n* = 1), H17 (*n* = 1), other HCFs (*n* = 2) and community sources (*n* = 4). Sub-cluster I isolates differed from each other by an average of 26 SNVs (range: 0–80 SNVs) (Fig 2). Isolates from H1 differed from each other by an average of 23 SNVs (range: 0–70 SNVs). Isolate M14/0992, recovered from a HCW in H1 and isolate M15/0213, recovered from another HCW in H1 who presented to a community-based general practitioner (GP), were both included in sub-cluster I. They differed from all other H1/sub-cluster I isolates by 12–53 SNVs and 7–47 SNVs, respectively. Sub-cluster I also included two isolate pairs, recovered from separate patients at different times, which exhibited intra-pair differences in susceptibility to mupirocin. Isolates M15/0148 (mupirocin-susceptible) and M15/0637 (Hi-MupR) from patient one were recovered nine months apart and differed by 33 SNVs, while isolates M15/0540 (mupirocin-susceptible) and M15/0541(Hi-MupR) from patient two were recovered 23 days apart and differed by four SNVs (Fig 2). The only environmental isolate investigated, which was recovered from hospital H1, was located in sub-cluster I. This differed from all other H1 isolates by 14–59 SNVs. The four CA isolates within sub-cluster I differed from H1/sub-cluster I isolates by 3–80 SNVs and from isolates from other hospitals/HCFs in sub-cluster I by 5–67 SNVs.

Sub-cluster II consisted of 19 isolates recovered over 36 months (June 2013–June 2016), including the remaining H1 isolates (3/46; 6.5%) and isolates from hospitals H2 (*n* = 2), H3 (*n* = 1), H4 (*n* = 2), H5 (*n* = 2), H8 (*n* = 1), H11 (*n* = 1), H 15 (*n* = 1), a nursing home (*n* = 1) and community sources (*n* = 5) (Fig 2). Isolates M14/0845 and M14/0857, recovered on the same day from different patients on the same ward of hospital H5, differed by two SNVs. The CA isolate M16/0002 and hospital H6 isolate M16/0116 were recovered 43 days apart and differed by six SNVs. Apart from these two instances, isolates within sub-cluster II differed from each other by 42–127 SNVs. The H1 isolates in sub-cluster II differed by 42–121 SNVs. The intra-cluster outlier isolates, M14/0597 and M16/0223, branched off from sub-cluster II (Fig 2). Both isolates were HCA and differed from sub-cluster II isolates by 91–149 and 79–128 SNVs, respectively.

The 11 isolates outside of the major MST cluster (outliers) were recovered over 29 months (October 2013–March 2016) and included one isolate from each of hospitals H2, H6, H9, H10, H12, H14 and H16, and two isolates from both hospital H7 and community sources (Fig 1). Two outlier isolates, M14/0994 and M14/0886 (Fig 1), were recovered 49 days apart from different patients on separate wards in hospital H7 and differed by two SNVs. Excluding this pair of isolates, the outliers differed from each other by 50–319 SNVs.

### High-level mupirocin resistance

Phenotypic high-level mupirocin-resistance was detected in 50/89 (56.2%) isolates, all of which were *ileS2*-positive and located in MST sub-cluster I (Fig 2). A total of 46/50 (92.0%) Hi-MupR isolates were HCA and 4/50 (8%) were CA. The majority of HCA Hi-MupR isolates (40/46; 87.0%) were from H1 and accounted for 87.0% (40/46) of all H1 outbreak isolates. The remaining 6/46 (8.7%) HCA Hi-MupR isolates were from hospitals H3 (*n* = 2), H4 (*n* = 1), H13 (*n* = 1), H17 (*n* = 1) and a long-term care facility (*n* = 1). DNA microarray profiling detected the *ileS2* gene in two phenotypically mupirocin-susceptible isolates (M15/0201 and M15/0540) within MST sub-cluster I. A single adenine insertion at nucleotide position 283 in *ileS2* was identified in each isolate, resulting in a downstream frameshift mutation and a premature stop codon. Twenty-three days after the recovery of isolate M15/0540, a phenotypically Hi-MupR isolate (M15/0541) harboring *ileS2* without the adenine insertion was recovered from the same patient (P1, Fig 2).

### A single *ileS2*-encoding plasmid in all Hi-MupR isolates

Mating and plasmid curing experiments using the Hi-MupR isolate M14/0355 confirmed the presence of a conjugative *ileS2*-encoding plasmid. DNA microarray profiling confirmed the gain of *ileS2* in transconjugants and its loss in cured derivatives, which were Hi-MupR (mupirocin MIC >1024 mg/L) and mupirocin-susceptible (mupirocin MIC <1 mg/L), respectively. A BLAST analysis of the single-molecule real-time (SMRT) derived sequence of the *ileS2*-encoding plasmid of M14/0355 revealed that it shared 99% DNA sequence identity with *iles2*-encoding plasmid, pV030-8 (GenBank accession number: NC_010279). Successful alignment of both the sequence reads and contigs of the remaining 49 Hi-MupR isolates to the SMRT sequence of the *ileS2*-encoding plasmid of M14/0355 confirmed the presence of the pV030-8-like plasmid in all 50 Hi-MupR isolates. The SMRT sequence of the M14/0355 *ileS2*-encoding plasmid (p140355) has been submitted to GenBank (accession number: KY465818).

### Carriage of SCC*fus*

DNA microarray profiling showed that SCC*fus*, encoding fusidic acid resistance, was only carried by the outlier isolates (7/11; 63.7%), both HCA (*n* = 6) and CA (*n* = 1) (Fig 1).

## Discussion

This study revealed the recent emergence and extensive spread of several strains of a predominantly MDR CA-MRSA clone, CC1-ST1-MRSA-IV, within and between hospitals/HCFs and communities throughout Ireland. Its resistance to many clinically relevant antibiotics (Table 1), which restricts patient treatment options and its often Hi-MupR nature, which eliminates the option of mupirocin nasal decolonization, merit particular concern. Interestingly, increased prevalence of a MDR CA-MRSA clone, *pvl*-positive CC1-ST772-MRSA-V, was previously reported in Ireland [22]. Although this clone did not spread as extensively as the CC1-ST1-MRSA-IV clone investigated here, this pattern reflects the relatively recent worldwide trend of CA-MRSA spreading into hospitals. Worryingly, the MDR CA-MRSA clone investigated here was detected in 17 hospitals, four other HCFs and from 11 people in the community, over the last three years (2013–2016). The extensive spread of this CA-MRSA clone within and between the Irish community and hospitals/HCFs highlights the need for infection prevention and control measures that consider CA-MRSA transmission routes into hospitals. While current Irish National Clinical Guidelines for infection prevention and control of MRSA recommend screening at-risk patients, only HCA-MRSA risk factors are considered [47]. Additionally, routine screening of HCWs, who are a potential source of CA-MRSA, is not mandatory in Ireland, except during outbreaks.

The use of cgMLST analysis grouped the majority of isolates (78/89) into one major MST cluster, leaving just 11 outliers. Pairwise SNV comparison subsequently provided enhanced discrimination between isolates. In order to inform our SNV comparison interpretation, we considered the SNV analysis data sets associated with multiple ST1-MRSA-IV isolates derived from single patient swabs, which indicated that a difference of ≤43 SNVs could be deemed negligible when assessing relatedness between ST1-MRSA-IV isolates. This maximum intra-host strain variation estimation conforms with that of 40 SNVs, previously established for *S*. *aureus* belonging to ST22, ST2257, ST30 and ST36 [15]. In some previous studies, this estimation has been used as a relatedness-threshold, with isolates differing by ≤40 SNVs being deemed closely related [10, 16]. However, given the external pressures to which isolates from the present study were presumably subjected during the three-year period in which they were recovered, this 40 SNV relatedness threshold was deemed inappropriate. Considering this, combined with the assumption that the MST indicated the most probable relationship between isolates, or at least the presence of isolate groups, the major MST cluster isolates (Fig 1), which differed by a maximum of 127 SNVs, were deemed closely related. Thus, the contemporaneous circulation of two closely related strains, and multiple sporadic strains, was identified (Fig 2).

Strain I isolates (sub-cluster I, Fig 2) were identified mainly in hospital H1 (43/46 isolates) but also in four other hospitals, two HCFs and from the community. Upon further investigation of strain I, it was found that H1 isolates differed from isolates from other hospitals/HCFs by as few as three SNVs and that the CA isolates differed from H1 isolates and isolates from other hospitals/HCFs by as few as three and five SNVs, respectively. Although definitive conclusions cannot be drawn regarding the original source(s) of this strain, these data clearly indicate that strain I spread within and between five different hospitals, two HCFs and the community. Interestingly, two H1 HCWs carried strain I isolates differing from other H1 isolates by as few as seven and 12 SNVs, respectively. It therefore cannot be ruled out that HCWs acted as a reservoir for this strain during the outbreak. Travel of staff between healthcare facilities is common in Ireland and this, combined with the transfer of patients between hospitals/HCFs, likely contributed to the dissemination of strain I. The use of mupirocin in hospital H1 may have driven selection of Hi-MupR strain I isolates. Hospitals in Ireland follow national guidelines for patients and healthcare staff found to be colonized with MRSA [47]. Attempts at decolonization may be considered for colonized patients who are due to undergo an elective operative procedure, patients in a clinical area where there is a high risk of colonization leading to invasive infection, if the risk of infection is high and the consequences severe (e.g. immunocompromised patients), or as part of a strategy to address uncontrolled transmission despite the use of other measures. National guidelines for MRSA decolonization recommend the use of nasal treatment with mupirocin and a chlorhexidine body wash. Interestingly, 53/57 (93%) strain I isolates also harbored *qac* genes encoding resistance to quaternary ammonium compounds such as chlorhexidine (S1 Table).

Strain II isolates (sub-cluster II, Fig 2) were recovered from eight different hospitals, one nursing home and the community. This strain, although represented by fewer isolates (strain I, *n* = 57; strain II, *n* = 19), was more divergent than strain I, exhibiting a higher SNV range (strain I, 0–80 SNVs; strain II, 0–127 SNVs). A difference of just six SNVs between H6 isolate, M16/0116, and CA isolate, M16/0002, indicated that strain II transmission between a nosocomial and community setting had occurred in at least one instance.

Eleven ST1-MRSA-IV-t127 isolates, not assigned to strain I or II (outlier isolates, Fig 1) were recovered from eight hospitals and the community in Ireland during the time period in which strains I and II circulated. It is possible that the predominantly MDR nature of strains I and II, lacking in the majority (10/11) of outlier isolates, may have facilitated their spread. Although rarely MDR, 63.6% of outlier isolates harbored SCC*fus* and exhibited fusidic acid resistance, suggesting that fusidic acid usage may have encouraged selection of these strains. While systemic use of fusidic acid has decreased in Ireland in recent years [48], topical use of fusidic acid in the community may have contributed towards selection of these strains.

The *ileS2*-encoding plasmid, p140355, carried by all Hi-MupR MRSA isolates exhibited 99% DNA sequence identity to the previously described pV030-8 plasmid, (GenBank accession number: NC_010279, direct submission) identified in 2007 in South Korea. Reports of this plasmid in the literature however, are lacking and its global prevelance is unknown. At 39 kb, p140355 is approximately 2.7 kb smaller than the more commonly reported *ileS2*-encoding pPR9 plasmid [49].

Several studies reported the displacement of previously predominant HCA- by CA-MRSA clones, highlighting the importance of continued monitoring and surveillance of CA-MRSA both in hospitals and communities. In India, the MDR CA and *pvl*-positive CC1-ST772-MRSA-V clone displaced the previously predominant HCA ST239-MRSA-III clone [8, 50]. In the USA, the CA ST8-MRSA-IV clone USA300 now constitutes the leading cause of MRSA nosocomial infections, having overtaken the previously predominant HCA ST5-MRSA-II clone, USA100 [51]. While ST22-MRSA-IV continues to predominate as the major cause of nosocomial MRSA infections in Ireland, ST1-MRSA-IV isolates represented the second most common clone identified by the NMRSARL in 2015 [46]. Hospital outbreaks involving ST1-MRSA have been reported elsewhere in Europe including the UK, Denmark and Italy in 2006, 2008 and 2012, respectively [52–54]. The emergence of ST1 MRSA in nosocomial settings is not exclusively confined to outbreak scenarios; in 2015 ST1-MRSA was the most common clone circulating in seven nursing homes in Shanghai, China, accounting for 29.1% of MRSA [31].

The emergence of a predominantly MDR CA-MRSA clone and its subsequent dissemination into hospitals, HCFs and the community throughout Ireland is worrying. Infection prevention and control measures should consider CA-MRSA risk factors and not only HCA-MRSA risk factors during MRSA screening and should recognize the importance of screening HCWs for MRSA.

## Materials and methods

### Ethics statement

None of the work described in this manuscript involved human subjects or work on animals. The study investigated MRSA isolates from patients submitted to NMRSARL. No patient identifying information whatsoever is contained in the manuscript.

### Isolates

In 2010, 0.18% of all MRSA isolates identified at the NMRSARL were mupirocin-susceptible MRSA-t127. However, in 2015 4.49% and 2.59% of all isolates identified at the NMRSARL were mupirocin-susceptible and Hi-MupR MRSA-t127, respectively. Eighty-seven *spa* type t127-MRSA and two *spa* type t922-MRSA isolates identified at the NMRSARL between June 2013 and June 2016 were investigated in the present study (S1 Table). All isolates were *pvl*-negative. Isolates were deemed to be HCA if they were recovered from hospital in-patients at least 48 h post-admission (*n* = 72), the hospital environment (*n* = 1), from hospital healthcare workers (*n* = 2) or from residents in nursing homes (*n* = 2) and long-term care facilities (*n* = 1). Isolates were deemed to be CA-MRSA if they were recovered from patients attending community-based GPs (*n* = 9) and hospital accident and emergency (*n* = 1) and outpatient departments (*n* = 1). Forty-six of the HCA isolates were recovered from colonized or infected sites of in-patients (*n* = 44), a colonized healthcare worker (*n* = 1) or from the environment (*n* = 1) in an 820-bed acute care hospital in Dublin, Ireland (H1) during a protracted t127-MRSA outbreak between November 2013 and February 2016 involving patients from 11 wards (29 isolates were from one ward (ward A)). Outbreak isolates were initially detected from clinical samples. Further cases were identified from screening specimens taken from patients with risk factors for MRSA colonization (such as previous history of MRSA colonization/infection at hospital H1) and from clinical samples. Following the identification of the outbreak, H1 in-patients in the affected areas were subjected to active screening for MRSA. Thirty-two of the HCA isolates were recovered between October 2013 and March 2016 from in-patients in 16 other hospitals (H2-H17) or from other HCFs. Only one isolate per patient was included in this study with the exception of three pairs of isolates from separate patients: patient one isolates, M15/0148 (H1, ward E; mupirocin-susceptible and *ileS2*-negative) and M15/0637 (recovered by patient’s GP; Hi-MupR and *ileS2*-positive); patient two isolates, M15/0540 (H1, ward A; phenotypically mupirocin-susceptible but *ileS2*-positive) and M15/0541 (H1, ward I; Hi-MupR and *ileS2*-positive), patient three isolates, M14/0965 (H1, ward A; Hi-MupR and *ileS2*-positive) and M15/0223 (H1, ward B; Hi-MupR and *ileS2*-positive). Isolates from patients one to three were recovered nine months, 23 days and four months apart, respectively (S1 Table). Furthermore, multiple colonies recovered from two separate patient swabs (same patients that yielded M13/0653 and M15/0221) were used to determine intra-strain variation *in vivo* (see below).

Isolates were identified as *S*. *aureus* using the tube coagulase test and methicillin resistance was detected using 30-μg cefoxitin disks (Oxoid Ltd., Basingstoke, United Kingdom) in accordance with European Society of Clinical Microbiology and Infectious Diseases (EUCAST) methodology and interpretive criteria [55]. MRSA isolates were stored at -80°C on individual Protect Bacterial Preservation System cryogenic beads (Technical Services Consultants Ltd., Heywood, United Kingdom).

### Antimicrobial susceptibility testing

The susceptibility of all isolates was determined against a panel of 23 antimicrobial agents and heavy metals by disk diffusion using EUCAST methodology and interpretative criteria [55]. If not available, Clinical Laboratory Standards Institute disk concentrations and interpretive criteria were used [56], or for the remaining agents (including all heavy metals tested), the disk concentrations and interpretive criteria of Rossney *et al*. were used [39]. The 23 agents tested were amikacin, ampicillin, cadmium acetate, chloramphenicol, clindamycin, ciprofloxacin, erythromycin, fusidic acid, gentamicin, kanamycin, linezolid, mercuric chloride, mupirocin, neomycin, phenyl mercuric acetate, rifampicin, spectinomycin, streptomycin, sulphonamide, tetracycline, tobramycin, trimethoprim and vancomycin.

The mupirocin MIC of each isolate was determined using mupirocin E-test strips (bioMérieux, Nuertlingen, Germany) according to the manufacturer’s instructions. Following incubation for 24 h at 37°C, the mupirocin MIC of each isolate was determined to be the nearest two-fold dilution, above which there was no visible growth. Isolates were deemed to be mupirocin susceptible if they exhibited a mupirocin MIC of ≤1 mg/L, to exhibit low-level mupirocin resistance if they had a mupirocin MIC of 2–128 mg/L, or to exhibit high-level mupirocin resistance if they exhibited a mupirocin MIC ≥ 256 mg/L [55].

### Molecular typing of isolates

All isolates underwent *spa* typing and DNA microarray profiling. For *spa* typing, genomic DNA was extracted from isolates using a 6% InstaGene matrix solution according to the manufacturer’s instruction (BioRad, München, Germany). Sequences were analyzed using the Ridom StaphType software package version 1.5 (Ridom Gmbh, Wurzburg, Germany) and *spa* types were assigned using the SpaServer website (http://spaserver2.ridom.de).

Genomic DNA for DNA microarray profiling was extracted from each isolate by enzymatic lysis using the buffers and solutions provided with the *S*. *aureus* Genotyping Kit 2.0 (Alere Technologies GmbH, Jena, Germany) and the DNeasy blood and tissue kit (Qiagen, Crawley, West Sussex, United Kingdom) according to the manufacturer's instructions. DNA microarray profiling was performed using the *S*. *aureus* Genotyping Kit 2.0 (Alere), which consists of individual DNA microarrays mounted in 8-well microtiter strips that detect 333 *S*. *aureus* gene sequences and alleles, including species-specific, antimicrobial resistance and virulence-associated genes, SCC*mec* genes and typing markers. ArrayMate software (version 2012-01-18) (Alere) was used to analyze data generated by the microarray system and to assign isolates to STs and/or CCs by comparing the microarray profile results of test isolates to the corresponding profiles of an extensive range of reference strains stored in the ArrayMate database that had previously undergone MLST [57]. The primers, probes, and protocols for the DNA microarray system have been described in detail previously [58].

### Plasmid conjugation and curing

The plasmid-free novobiocin-resistant *S*. *aureus* laboratory strain XU21 was used as a plasmid recipient strain during filter mating experiments [59]. Conjugative transfer of the plasmid-encoded *ileS2* gene from the t127-MRSA isolate M14/0355 to the plasmid-free *S*. *aureus* recipient strain XU21 was performed by filter mating as described previously [59]. Presumptive transconjugant derivatives were selected by subculture on brain heart infusion (BHI) agar (Oxoid Ltd.) supplemented with mupirocin (100 μg/ml) (GlaxoSmithKline, Citywest Business Campus, Dublin, Ireland) and novobiocin (10 μg/ml) (Sigma-Aldrich) and were confirmed by DNA microarray profiling and mupirocin MIC determination.

Curing of the *ileS2*-encoded plasmid from isolate M14/0355 was performed following reactivation from a cryogenic bead on to a Tryptic Soy Agar plate and culturing one colony in 5 ml of Brain Heart Infusion (BHI) broth (Oxoid Ltd.) at 43°C and 200 rpm for 24 h. This was followed by subculturing 0.1 ml into 5 ml of fresh BHI broth and incubation as before (43°C and 200 rpm for 24 h) for four consecutive rounds. Individual colonies obtained following plating on BHI agar were screened for the loss of mupirocin resistance by replica plating onto BHI agar supplemented with mupirocin at 100 μg/ml and putative cured derivatives were confirmed by DNA microarray analysis.

### Whole-genome sequencing

Genomic DNA for whole-genome sequencing was extracted using the Qiagen DNeasy blood and tissue kit according to the manufacturer's instructions. Whole-genome sequencing was undertaken using the Nextera XT library preparation reagents in accordance with the manufacturer’s instruction (Illumina, Eindhoven, The Netherlands). Libraries were sequenced on an Illumina MiSeq instrument. Resulting fastQ files were imported directly from Illumina BaseSpace to the BioNumerics (version 7.6) (Applied Maths, Belgium) cloud-based calculation engine, where they were assembled using the Velvet *de novo* genome assembler (version 1.2.10). Both the fastQ files and assembled genome of each isolate were submitted to the BioNumerics wgMLST scheme for assembly-free and assembly-based allele calling, respectively. To investigate relationships between isolates, a MST was generated using BioNumerics, based on core-genome loci, as previously described by Leopold *et al*. [18]. The genome of the centrally located isolate in the MST, M15/0029 (designated as the “root”) (Fig 1), was chosen as the reference sequence against which all other isolate genomes were mapped. The BioNumerics genome analysis tool was used to record SNVs between each isolate and the root, yielding a SNV matrix detailing all SNV positions in the pan genome. Using Clustal Omega, a multiple sequence alignment of the SNV matirix was carried out and an n × n percentange identity matrix was generated [60]. In order to calculate SNVs between all possible isolates pairs, a pairwise SNV matrix was created (S1 Dataset) by applying the following equation to the percentage identity matrix:

$$x=\frac{\left(100-y)(n\right)}{100}$$

Where, *x* = the number of SNVs by which two isolates differ

*y* = the percentage identity of the SNV matrix sequence of two isolates

*n* = the total number of SNV positions in the pan genome

The ST of each isolate was also assigned using WGS data and the Ridom SeqSphere+ software package version 3.3.0 (Ridom GmbH, Germany).

### Plasmid sequence analysis using WGS data

The Hi-MupR ST1-MRSA-IV-t127 isolate that underwent conjugation and curing (M14/0355) also underwent SMRT sequencing (Pacific Biosciences, Norwich, United Kingdom) in order to obtain the nucleotide sequence of the entire *ileS2*-containing plasmid on one contiguous sequence. The MiSeq-generated reads of the remaining Hi-MupR MRSA isolates were mapped to the M14/0355 SMRT sequence using the Burrows-Wheeler aligner (BWA-mem) (http://arxiv.org/abs/1303.3997). Artemis sequence viewer (http://www.sanger.ac.uk/science/tools/artemis) was used to visually assess the mapping of reads to the M14/0355 sequence. Contigs were generated by *de novo* assembly using the SPAdes assembler [61], were aligned to the M14/0355 SMRT sequence using the BWA-mem and were visualized using Artemis. Blast was used to search the literature for *ileS2*-encoding plasmids similar to that harbored by M14/0355 (https://www.blast.ncbi.nlm.nih.gov).

### *In vivo* SNV investigation

In order to inform the interpretation of the WGS SNV data, the SNVs of two sets of 13 individual colonies cultured from separate patient swabs (the patients from which study isolates M13/0653 and M15/0221 were recovered, respectively) were investigated. Colonies were recovered from both swabs by plating on SaSelect chromogenic agar plates (BioRad) for isolation and initial identification of *S*. *aureus* following incubation at 37°C for 24 h. Following incubation, 13 well separated individual colonies were each subcultured onto separate SaSelect chromogenic agar plates to obtain pure cultures. In each case, following confirmation of *S*. *aureus* identification by latex agglutination using the Pastorex™ Staph-Plus kit (Bio-Rad, Hercules, California, USA), one colony from each of the 13 plates was selected for *spa* typing and DNA microarray profiling. Genomic DNA was extracted using the Qiagen DNeasy blood and tissue kit and underwent WGS preparation and MiSeq sequencing, as described above. The BioNumerics genome analysis tool was used to record SNVs between the genomes of each colony, yielding a pan genome SNV matrix that was analyzed in order to generate a pairwise SNV matrix (S2 and S3 Datasets).

### Accession number

The nucleotide sequence of the *ileS2*-encoding plasmid (p140355) from ST1-MRSA-IV-t127/t922 isolate M14/0355 has been submitted to GenBank (accession number: KY465818).

## Supporting information

S1 Dataset Pairwise SNV matrix of all 89 ST1-MRSA-IV isolates used to infer relatedness between isolates. (XLSX)

S2 Dataset Pairwise SNV matrix—swab (A). Pairwise SNV matrix of 13 colonies from a single swab (from the same patient from which isolate M13/0653 was recovered) used to determine the maximum intra-host variation of an ST1-MRSA-IV isolate and inform interpretation of the ST1 pairwise SNV matrix.
(XLSX)

S3 Dataset Pairwise SNV matrix—swab (B). Pairwise SNV matrix of 13 colonies from a single swab (from the same patient from which isolate M15/0221 was recovered) used to determine the maximum intra-host variation of an ST1-MRSA-IV isolate and inform interpretation of the ST1 pairwise SNV matrix.
(XLSX)

S1 Table Isolate information. Epidemiological, phenotypic and molecular characteristics of t127 and t922 methicillin-resistant *Staphylococcus aureus* isolates recovered from 17 hospitals, four other healthcare facilities and the community throughout Ireland between 2013 and 2016.
(PDF)
