# Supplementary material for: The recent emergence in hospitals of multidrug-resistant community-associated sequence type 1 and spa type t127 methicillin-resistant Staphylococcus aureus investigated by whole-genome sequencing: Implications for screening
Source: PLoS One. 2017 Apr 11;12(4):e0175542. doi: 10.1371/journal.pone.0175542 (PMC5388477; doi:10.1371/journal.pone.0175542)
Supplement: S1 Table — Epidemiological, phenotypic and molecular characteristics of t127 and t922 methicillin-resistant Staphylococcus aureus isolates recovered from 17 hospitals, four other healthcare facilities and the community throughout Ireland between 2013 and 2016. (PDF) [file pone.0175542.s004.pdf]

S1 Table. Isolate information[a]

Location of patient	Hospital ward	Isolate number	Date of recovery	Source	Clinical presentation	Sequence type (ST)-SCC*mec* type [b]	*spa* type	Antimicrobial resistance profile[c]	Antimicrobial resistance and virulence-associated genes[d]
H1	A	M13/0653	11/05/2013	HCA	Colonization	ST1-IV	t127	AMP, ERY, KAN, MUP, NEO, STR, TET, TOB	*blaZ*, *erm*(C), *lnu*(A), *vga*, *aadD*, *aphA3*, *sat*, *mupA*, *tet*(K), *sdrM*, *seh*, IEC E (*sak*, *scn*)
		M14/0373	05/11/2014	HCA	Colonization	ST1-IV	t127	AMP, ERY, KAN, MUP, NEO, STR, TET	*blaZ*, *erm*(C), *aphA3*, *sat*, *mupA*, *tet*(K), *qacA*, *sdrM*, *seh*, IEC E (*sak*, *scn*)
		M14/0279	05/19/2014	HCA	SSTI	ST1-IV	t127	AMP, ERY, KAN, MUP, NEO, STR, TET	*blaZ*, *erm*(C), *aphA3*, *sat*, *mupA*, *tet*(K), *fosB, qacB*, *qacA*, *sdrM*, *seh*, IEC E (*sak*, *scn*)
		M13/0671	11/15/2013	HCA	Colonization	ST1-IV	t127	AMP, ERY, KAN, MUP, NEO, STR, TET	*blaZ*, *erm*(C), *aphA3*, *sat*, *mupA*, *tet*(K), *qacA*, *sdrM*, *seh*, IEC E (*sak*, *scn*)
		M14/0355	05/30/2014	HCA	SSTI	ST1-IV	t127	AMP, ERY, KAN, MUP, NEO, STR, TET	*blaZ*, *erm*(C), *vga*, *aphA3*, *sat*, *mupA*, *tet*(K), *qacA*, *sdrM*, *seh*, IEC E (*sak*, *scn*)
		M14/0425	06/24/2014	HCA	Colonization	ST1-IV	t127	AMP, ERY, KAN, MUP, NEO, STR, TET	*blaZ*, *erm*(C), *vga*, *aphA3*, *sat*, *mupA*, *tet*(K), *qacB*, *fosB*, *qacA*, *sdrM*, *seh*, IEC E (*sak*, *scn*)
		M14/0480	07/01/2014	HCA	Colonization	ST1-IV	t127	AMP, ERY, KAN, MUP, NEO, STR, TET	*blaZ*, *erm*(C), *aphA3*, *sat*, *mupA*, *tet*(K), *qacA*, *sdrM*, *seh*, IEC

							E (*sak*, *scn*)
M14/0481	07/01/2014	HCA	Colonization	ST1-IV	t127	AMP, ERY, KAN, MUP, NEO, STR, TET	*blaZ*, *erm*(C), *vga*, *aphA3*, *sat*, *mupA*, *tet*(K), *qacA*, *qacB*, *sdrM*, *seh*, IEC E (*sak*, *scn*)
M14/0466	07/02/2014	HCA	Colonization	ST1-IV	t127	AMP, KAN, NEO, ERY, STR, TET, MUP	*blaZ*, *aphA3*, *sat*, *mupA*, *sdr*(M) *seh*, *sak*, *scn*, *qacA*
M15/0068	01/20/2015	HCA	BSI	ST1-IV	t127	AMP, ERY, KAN, MUP, NEO, STR, TET	*blaZ*, *erm*(C), *aphA3*, *sat*, *mupA*, *tet*(K), *qacA*, *sdrM*, *seh*, IEC E (*sak*, *scn*)
M14/0586	07/08/2014	HCA	Colonization	ST1-IV	t127	AMP, ERY, KAN, MUP, NEO, STR, TET	*blaZ*, *erm*(C), *aphA3*, *sat*, *mupA*, *tet*(K), *qacA*, *sdrM*, *seh*, IEC E (*sak*, *scn*)
M14/0602	07/16/2014	HCA	Colonization	ST1-IV	t127	AMP, ERY, KAN, MUP, NEO, PMA, STR, TET	*blaZ*, *erm*(C), *aphA3*, *sat*, *mupA*, *tet*(K), *qacA*, *sdrM*, *seh*, IEC E (*sak*, *scn*)
M14/0603	07/16/2014	HCA	Colonization	ST1-IV	t127	AMP, ERY, KAN, MUP, NEO, STR, TET	*blaZ*, *erm*(C), *aphA3*, *sat*, *mupA*, *tet*(K), *qacA*, *sdrM*, *seh*, IEC E (*sak*, *scn*)
M14/0656	07/28/2014	HCA	Colonization	ST1-IV	t127	AMP, ERY, KAN, MUP, NEO, STR, TET	*blaZ*, *erm*(C), *aphA3*, *sat*, *mupA*, *tet*(K), *qacA*, *sdrM*, *seh*, IEC E (*sak*, *scn*)
M14/0660	07/28/2014	HCA	Colonization	ST1-IV	t127	AMP, CDA, ERY, KAN, MUP, NEO, STR, TET	*blaZ*, *erm*(C), *aphA3*, *sat*, *mupA*, *tet*(K), *qacA*, *sdrM*, *seh*, IEC E (*sak*, *scn*)
M14/0664	08/05/2014	HCA	Colonization	ST1-IV	t127	AMP, CDA, ERY, KAN, MUP, NEO,	*blaZ*, *erm*(C), *aphA3*, *sat*, *mupA*, *qacA*, *sdrM*, *seh*, IEC E (*sak*,

						STR, TET	*scn*)
M14/0681	08/15/2014	HCA	SSTI	ST1-IV	t127	AMP, ERY, KAN, MUP, NEO, STR, TET	*blaZ*, *erm*(C), *aphA3*, *sat*, *mupA*, *qacA*, *sdrM*, *seh*, IEC E (*sak*, *scn*)
M14/0695	08/21/2014	HCA	Colonization	ST1-IV	t127	AMP, ERY, KAN, MUP, NEO, STR, TET	*blaZ*, *erm*(C), *aphA3*, *sat*, *mupA*, *qacA*, *sdrM*, *seh*, IEC E (*sak*, *scn*)
M14/0868	10/16/2014	HCA	Colonization	ST1-IV	t127	AMP, CDA, ERY, KAN, MUP, NEO STR, TET	*blaZ*, *erm*(C), *aphA3*, *sat*, *mupA*, *qacA*, *sdrM*, *seh*, IEC E (*sak*, *scn*)
M14/0876	10/22/2014	HCA	Colonization	ST1-IV	t127	AMP, CDA, ERY, KAN, MUP, NEO, STR, TET	*blaZ*, *erm*(C), *aphA3*, *sat*, *mupA*, *qacA*, *sdrM*, *seh*, IEC E (*sak*, *scn*)
M14/0892	10/22/2014	HCA	Colonization	ST1-IV	t127	AMP, CDA, ERY, KAN, MUP, NEO, STR, TET	*blaZ*, *erm*(C), *aphA3*, *sat*, *mupA*, *tet*(K), *qacA*, *sdrM*, *seh*, IEC E (*sak*, *scn*)
M14/0878	10/23/2014	HCA	Colonization	ST1-IV	t922	AMP, CDA, ERY, KAN, MUP, NEO, STR, TET	*blaZ*, *erm*(C), *aphA3*, *sat*, *mupA*, *tet*(K), *qacA*, *sdrM*, *seh*, IEC E (*sak*, *scn*)
M14/0877	10/27/2014	HCA	Colonization	ST1-IV	t127	AMP, CDA, ERY, KAN, MUP ,NEO, STR, TET	*blaZ*, *erm*(C), *aphA3*, *sat*, *mupA*, *tet*(K), *qacA*, *sdrM*, *seh*, IEC E (*sak*, *scn*)
M14/0966	11/17/2014	HCA	Colonization	ST1-IV	t127	AMP, ERY, KAN, MUP, NEO, STR, TET	*blaZ*, *erm*(C), *aphA3*, *sat*, *mupA*, *tet*(K), *qacA*, *sdrM*, *seh*, IEC E (*sak*, *scn*)
M14/0965[e]	11/25/2014	HCA	Graft infection	ST1-IV	t127	AMP, ERY, KAN, MUP, NEO, STR, TET	*blaZ*, *erm*(C), *aphA3*, *sat*, *mupA*, *qacA*, *sdrM*, *seh*, IEC E (*sak*,

								scn)
	M15/0149	01/25/2015	HCA	Colonization	ST1-IV	t127	AMP, CDA , ERY, KAN, NEO, STR, TET	*blaZ*, *erm*(C), *aphA3*, *sat*, *tet*(K), *sdrM*, *seh*, IEC E (*sak*, *scn*)
	M15/0540[f]	07/31/2015	HCA	Colonization	ST1-IV	t127	AMP, ERY, KAN, NEO, STR, TET	*blaZ*, *erm*(C), *aphA3*, *sat*, *mupA*, *tet*(K), *qacA*, *sdrM*, *seh*, IEC E (*sak*, *scn*)
	M14/0125	02/26/2014	HCA	Colonization	ST1-IV	t127	AMP, ERY, KAN, MUP, NEO, STR, TET	*blaZ*, *erm*(C), *aphA3*, *sat*, *mupA*, tet(K), *qacA*, *sdrM*, *seh*, IEC E (*sak*, *scn*)
	M14/0992	12/19/2014	HCA-HCW	Colonization	ST1-IV	t127	AMP, ERY, KAN, NEO, STR, TET	*blaZ*, *erm*(C), *aphA3*, *sat*, *tet*(K), *sdrM*, *seh*, IEC E (*sak*, *scn*)
B	M15/0138	02/11/2015	HCA	Colonization	ST1-IV	t127	AMP, CDA , ERY, KAN, MUP, NEO, STR, TET	*blaZ*, *erm*(C), *aphA3*, *sat*, *mupA*, *tet*(K), *qacA*, *sdrM*, *seh*, IEC E (*sak*, *scn*)
	M15/0206	03/10/2015	HCA	Colonization	ST45-IV	t127	AMP, CDA , ERY, KAN, MUP, NEO, STR, TET	*blaZ*, *erm*(C), *aphA3*, *sat*, *mupA*, *qacA*, *sdrM*, *seh*, IEC E (*sak*, *scn*)
	M15/0223[e]	03/24/2015	HCA	Graft infection	ST1-IV	t127	AMP, CDA, ERY, KAN, MUP, NEO, STR, TET	*blaZ*, *erm*(C), *aphA3*, *sat*, *mupA*, *qacA*, *sdrM*, *seh*, IEC E (*sak*, *scn*)
	M15/0222	03/24/2015	HCA	Colonization	ST1-IV	t127	AMP, CDA, ERY, KAN, MUP, NEO, STR, TET	*blaZ*, *erm*(C), *aphA3*, *sat*, *mupA*, *tet*(K), *qacA*, *sdrM*, *seh*, IEC E (*sak*, *scn*)
C	M15/0029	11/15/2013	HCA	Colonization	ST1-IV	t127	AMP, CDA, ERY, KAN, NEO, STR, TOB	*blaZ*, *erm*(C), *aphA3*, *sat*, *tet*(K), *sdrM*, *seh*, IEC E (*sak*, *scn*)
	M15/0614	09/10/2015	HCA	BSI	ST1-IV	t127	AMP, ERY, FUS, KAN, MUP,	*blaZ*, *erm*(C), *aphA3*, *sat*, *fusB*, *mupA*,

							NEO, STR, TET	*qacC*, *sdrM*, *seh*, IEC E (*sak*, *scn*)
D	M14/0103	02/16/2014	HCA	Osteomylitis	ST1-IV	t127	AMP, ERY, KAN, MUP, NEO, SPT, STR, TET	*blaZ*, *erm*(C), *vgaA*, *aphA3*, *sat*, *mupA*, *tet*(K), *fosB*, *qacA*, *sdrM*, *seh*, IEC E (*sak*, *scn*)
	M14/0967	10/28/2014	HCA	Colonization	ST1-IV	t127	AMP, ERY, KAN, MUP, NEO, STR, TET	*blaZ*, *erm*(C), *aphA3*, *sat*, *mupA*, *tet*(K), *qacA*, *sdrM*, *seh*, IEC E (*sak*, *scn*)
E	M15/0148[g]	01/04/2015	HCA	Colonization	ST1-IV	t127	AMP, CDA, ERY, KAN, NEO, STR, TET	*blaZ*, *erm*(C), *aphA3*, *sat*, *tet*(K), *sdrM*, *seh*, IEC E (*sak*, *scn*)
	M15/0154	02/16/2015	HCA	Colonization	ST1-IV	t922	AMP, CDA, ERY, KAN, MUP, NEO, STR, TET	*blaZ*, *erm*(C), *aphA3*, *sat*, *mupA*, *tet*(K), *qacA*, *sdrM*, *seh*, IEC E (*sak*, *scn*)
F	M15/0221	03/17/2015	HCA	Unknown	ST1-IV	t127	AMP, CDA , ERY, KAN, MUP, NEO, STR, TET	*blaZ*, *erm*(C), *aphA3*, *sat*, *mupA*, *qacA*, *sdrM*, *seh*, IEC E (*sak*, *scn*)
G	M16/0141	02/06/2016	HCA	Colonization	ST1-IV	t127	AMP, ERY, KAN, NEO, MUP, STR	*blaZ*, *aphA3*, *sat*, *mupA*, *qacA*, *sdrM*, *seh*, IEC E (*sak*, *scn*)
H	M15/0724	12/04/2015	HCA	Colonization	ST1-IV	t127	AMP, ERY, KAN, MUP, NEO, STR, TET	*blaZ*, *erm*(C), *aphA3*, *sat*, *mupA*, *tet*(K), *qacA*, *sdrM*, *seh*, IEC E (*sak*, *scn*)
I	M15/0541[f]	08/23/2015	HCA	Colonization	ST1-IV	t127	AMP, ERY, KAN, MUP, NEO, STR, TET	*blaZ*, *erm*(C), *aphA3*, *sat*, *mupA*, *qacA*, *sdrM*, *seh*, IEC E (*sak*, *scn*)
J	M15/0030	02/25/2014	HCA	Colonization	ST1-IV	t127	AMP, CDA , ERY, KAN,	*blaZ*, *erm*(C), *aphA3*, *sat*, *tet*(K), *sdrM*, *seh*,

								NEO, STR, TOB	IEC E (*sak*, *scn*)
	K	M15/0337	05/27/2015	HCA	SSTI	ST1-IV	t127	AMP, CDA , ERY, KAN, MUP, NEO, STR, TET	*blaZ*, *erm*(C), *aphA3*, *sat*, *mupA*, *qacA*, *sdrM*, *seh*, IEC E (*sak*, *scn*)
		M14/0713	Unknown	Environ-ment	N/A	ST1-IV	t127	AMP, ERY, KAN, MUP, NEO, STR, TET	*blaZ*, *erm*(C), *aphA3*, *sat*, *mupA*, *tet*(K), *qacA*, *sdrM*, *seh*, IEC E (*sak*, *scn*)
H2	A	M15/0164	01/13/2015	HCA	Unknown	ST1-IV	t127	AMP, ERY, KAN, NEO, STR, TET	*blaZ*, *erm*(C), *aphA3, sat, qacA*, *sdrM, seh,* IEC E (*sak*, *scn*)
		M16/0188	03/08/2016	HCA	Unknown	ST1-IV	t127	AMP, ERY	*blaZ*, *erm*(A), *sdrM*, *seh*, IEC E (*sak*, *scn*)
	B	M14/0467	05/10/2014	HCA	Unknown	ST1-IV	t127	AMP, CDA, KAN, NEO, STR, TET	*blaZ*, *erm*(C), *aphA3*, *sat*, *sdrM*, *seh*, IEC E (*sak*, *scn*)
		M14/0697	08/20/2014	HCA	Unknown	ST1-IV	t127	AMP, CDA, FUS	*blaZ*, *sdrM*, *seh*, IEC E (*sak*, *scn*)
H3	A	M14/0993[h]	12/02/2014	HCA	BSI	ST1-IV	t127	AMP, ERY, FUS, KAN, MUP, NEO, STR, TET	*blaZ*, *erm*(C), *aphA3*, *sat*, *fusB*, *mupA*, *tet*(K), *qacA*, *sdrM*, *seh*, IEC E (*sak*, *scn*)
		M16/0139	02/09/2016	HCA	Unknown	ST1-IV	t127	AMP, ERY, KAN, NEO, MUP, STR, TET	*blaZ*, *erm*(C), *aphA3*, *sat*, *mupA*, *qacA*, *sdrM*, *seh*, IEC E (*sak*, *scn*)
	B	M15/0201	08/22/2014	HCA	Unknown	ST1-IV	t127	AMP, ERY, KAN, NEO, STR, TET	*blaZ*, *erm*(C), *aphA3*, *sat*, *mupA*, *qacA*, *sdrM*, *seh*, IEC E (*sak*, *scn*)
	C	M15/0307	05/11/2015	HCA	Unknown	ST1-IV	t127	AMP, ERY, KAN, NEO,	*blaZ*, *erm*(C), *aphA3*, *sat*, *sdrM*, *seh*, IEC E

								STR, TET	(*sak*, *scn*)
	D	M15/0127	08/18/2014	HCA	Unknown	ST1-IV	t127	AMP, ERY, FUS, KAN, NEO, STR	*blaZ*, *erm*(C), *aphA3*, *sat*, *qacA*, *sdrM*, *seh*, IEC E (*sak*, *scn*)
H4	A	M15/0286	04/30/2015	HCA	Unknown	ST1-IV	t127	AMP, ERY, KAN, NEO, STR, TET	*blaZ*, *erm*(C), *aphA3*, *sat*, *sdrM*, *seh*, IEC E (*sak*, *scn*)
	B	M15/0575	09/21/2015	HCA	Unknown	ST1-IV	t127	AMP, ERY, KAN, NEO, MUP, STR, TET	*blaZ*, *aphA3*, *sat*, *mupA*, *qacA*, *sdrM*, *seh*, IEC E (*sak*, *scn*)
	C	M15/0659	10/20/2015	HCA	Unknown	ST1-IV	t127	AMP, ERY, KAN, NEO, STR, TET	*blaZ*, *aphA3*, *sat*, *sdrM*, *seh*, IEC E (*sak*, *scn*)
H5	A	M14/0845	09/23/2014	HCA	Colonization	ST1-IV	t127	AMP, CDA, ERY, KAN, NEO, STR, TET	*blaZ*, *erm*(C), *aphA3*, *sat*, *tet*(K), *sdrM*, *seh*, IEC E (*sak*, *scn*)
		M14/0857	09/23/2014	HCA	Colonization	ST1-IV	t127	AMP, ERY, KAN, NEO, STR, TET	*blaZ*, *erm*(C), *aphA3*, *sat*, *sdrM*, *seh*, IEC E (*sak*, *scn*)
H6	A	M16/0123	01/08/2016	HCA	Unknown	ST1-IV/SCC*fus*	t127	AMP, ERY, FUS, TET	*erm*(C), *fusC*, *tet*(M), *sdrM*, *seh*, IEC E (*sak*, *scn*)
	Unknown	M16/0223	03/31/2016	HCA	Cellulitis, bursitis	ST1-IV	t127	AMP, ERY, KAN, NEO, STR, TET	*blaZ*, *erm*(C), *aphA3*, *sat*, *qacA*, *sdrM*, *seh*, IEC E (*sak*, *scn*)
H7	A	M14/0994	12/17/2014	HCA	Unknown	ST1-IV/SCC*fus*	t127	AMP, FUS	*blaZ*, *fusC*, *sdrM*, *seh*, IEC E (*sak*, *scn*)
	B	M14/0886	10/29/2014	HCA	Unknown	ST1-IV/SCC*fus*	t127	AMP, FUS	*blaZ*, *fusC*, *sdrM*, *seh*, IEC E (*sak*, *scn*)

H8	A	M16/0116	02/05/2016	HCA	Colonization	ST1-IV	t127	AMP, ERY, KAN, NEO, STR, TET	*blaZ*, *erm*(C), *aphA3*, *sat*, *sdrM*, *seh*, IEC E (*sak*, *scn*)
H9	A	M15/0640	10/14/2015	HCA	Unknown	ST1-IV	t127	AMP, CDA	*blaZ*, *sdrM*, *seh*, IEC E (*sak*, *scn*)
H10	A	M15/0161	02/06/2015	HCA	Colonization	ST1-IV/SCC*fus*	t127	AMP, CDA, ERY, FUS, SPT	*blaZ*, *erm*(C), *fusC*, *sdrM*, *seh*, IEC E (*sak*, *scn*)
H11	A	M15/0384	06/17/2015	HCA	Unknown	ST1-IV	t127	AMP, ERY, KAN, NEO, STR, TET	*blaZ*, *aphA3*, *sat*, *sdrM*, *seh*, IEC E (*sak*, *scn*)
H12	A	M15/0067	01/28/2015	HCA	Colonization	ST1-IV	t127	AMP, CDA, ERY	*blaZ*, *erm*(C), *sdrM*, *seh*, IEC E (*sak*, *scn*)
H13	Unknown	M15/0266	04/01/2015	HCA	Unknown	ST1-IV	t127	AMP, ERY, KAN, NEO, MUP, STR, TET	*blaZ*, *erm*(C), *aphA3*, *sat*, *mupA*, *sdrM*, *qacA*, *seh*, IEC E (*sak*, *scn*)
H14	A	M14/0953	11/27/2014	HCA	BSI	ST1-IV/SCC*fus*	t127	AMP, FUS	*blaZ*, *fusC*, *sdrM*, *seh*, IEC E (*sak*, *scn*)
H15	A	M15/0443	07/13/2015	HCA	Unknown	ST1-IV	t127	AMP, ERY, KAN, NEO, STR, TET	*blaZ*, *aphA3*, *sat*, *sdrM*, *seh*, IEC E (*sak*, *scn*)
H16	Unknown	M14/0015	10/24/2013	HCA	BSI	ST1-IV/SCC*fus*	t127	AMP, ERY, FUS	*erm*(C), *fusC*, *sdrM*, *seh*, IEC E (*sak*, *scn*)
H17	A	M14/0968[h]	11/25/2014	HCA	Colonization	ST1-IV	t127	AMP, ERY, KAN, MUP, NEO, STR, TET	*blaZ*, *erm*(*C*), *aphA3*, *sat*, *mupA*, *qacA*, *sdrM*, *seh*, IEC E (*sak*, *scn*)
NH	N/A	M15/0031[h]	05/01/2014	HCA	Colonization	ST1-IV	t127	AMP, CDA , ERY, KAN,	*blaZ*, *erm*(*C*), *aphA3*, *sat*, *tet*(*K*), *sdrM*, *seh*,

								NEO, STR, TOB	IEC E (*sak*, *scn*)
NH	N/A	M15/0609	09/25/2015	HCA	Unknown	ST1-IV	t127	AMP, CDA, ERY, KAN, NEO, STR, TET	*blaZ*, *aphA3*, *sat*, *qacA*, *sdrM*, *seh*, IEC E (*sak*, *scn*)
OHD	N/A	M14/0597	07/14/2014	HCA-HCW	Unknown	ST1-IV	t127	AMP, ERY, KAN, STR, TET	*blaZ*, *aphA3*, *sat*, *sdrM*, *seh*, IEC E (*sak*, *scn*)
LTCF	N/A	M15/0429[h]	07/06/2015	HCA	Unknown	ST1-IV	t127	AMP, CDA , ERY, KAN, MUP, NEO, STR, TET	*blaZ*, *erm*(*C*), *aphA3*, *sat*, *mupA*, *tet*(*K*), *qacA*, *sdrM*, *seh*, IEC E (*sak*, *scn*)
ED	N/A	M16/0183	03/02/2015	CA	Cellulitis, sepsis	ST1-IV	t127	AMP	*blaZ*, *sdrM*, IEC E (*sak*, *scn*)
OPD	N/A	M15/0371	06/04/2015	CA	Unknown	ST1-IV	t127	AMP, ERY, KAN, NEO, STR, TET	*blaZ*, *erm*(C), *aphA3*, *sat*, *sdrM*, *seh*, IEC E (*sak*, *scn*)
GP	N/A	M14/0046	05/11/2014	CA	Colonization	ST1-IV	t127	AMP, ERY, KAN, MUP, NEO, STR, TET	*blaZ*, *erm*(*C*), *aphA3*, *sat*, *mupA*, *tet*(*K*), *qacA*, *sdrM*, *seh*, IEC E (*sak*, *scn*)
GP	N/A	M14/0648	07/25/2014	CA	SSTI	ST1-IV/SCC*fus*	t127	AMP, FUS	*blaZ*, *fusC*, *sdrM*, *seh*, IEC E (*sak*, *scn*)
GP	N/A	M15/0245	04/01/2015	CA	SSTI	ST1-IV	t127	AMP, CDA , ERY, KAN, NEO, STR, TET	*blaZ*, *erm*(*C*), *aphA3*, *sat*, *sdrM*, *seh*
GP	N/A	M15/0382	06/17/2016	CA	SSTI	ST1-IV	t127	AMP, ERY, KAN, NEO, STR, TET	*blaZ*, *erm*(*C*), *aphA3*, *sat*, *sdrM*, *seh*, IEC E (*sak*, *scn*)
GP	N/A	M15/0637[g,h]	09/10/2015	CA	Colonization	ST1-IV	t127	AMP, ERY, KAN, MUP, NEO, STR, TET	*blaZ*, *erm*(*C*), *aphA3*, *sat*, *mupA*, *tet*(*K*), *qacA*, *sdrM*, *seh*, IEC

									E (*sak*, *scn*)
GP	N/A	M13/0404	06/13/2013	CA	SSTI	ST1-IV	t127	AMP, ERY, KAN, NEO, STR, TET	*blaZ*, *erm*(*C*), *aphA3*, *sat*, *tet*(*K*), *sdrM*, *seh*, IEC E (*sak*, *scn*)
GP	N/A	M16/0219	03/31/2016	CA	SSTI	ST1-IV	t127	AMP, KAN, NEO, MUP, STR, TET	*blaZ*, *aphA3*, *sat*, *mupA*, *qacA*, *sdrM*, *seh*, IEC E (*sak*, *scn*)
GP	N/A	M16/0002	12/24/2015	CA	SSTI	ST1-IV	t127	AMP, ERY, KAN, NEO, STR, TET	*blaZ*, *aphA3*, *sat*, *sdrM*, *seh*, IEC E (*sak*, *scn*)
GP	N/A	M15/0213[i]	03/16/2015	CA	SSTI	ST1-IV	t127	AMP, ERY, KAN, NEO, MUP, STR, TET	*erm*(C), *aphA3, sat, mupA, qacA*, *sdrM*, *seh*, IEC E (*sak*, *scn*)

Epidemiological, phenotypic and molecular characteristics of t127 and t922 methicillin-resistant *Staphylococcus* isolates recovered from 17 hospitals, four other healthcare facilities and the community throughout Ireland between 2013 and 2016.

[a] Isolates were selected for inclusion in this study if they exhibited *spa* type t127 (*spa* repeat succession: 07-23-21-16-34-33-13) or the closely related *spa* type t922 (07-23-21-16-33-13).

b Sequence types (STs) were determined by multilocus sequence typing using the Ridom SeqSphere software package version 3.3.0 (Ridom GmbH, Münster, Germany).

[c] Antimicrobial resistance phenotypes were determined by testing the susceptibility of isolates to a panel of 23 antimicrobial agents including amikacin, ampicillin (AMP), cadimium acetate (CDA), chloramphenicol, ciprofloxacin, clindamycin, erythromycin (ERY), fusidic acid (FUS), gentamicin, kanamycin (KAN), linezolid, mercuric chloride, mupirocin (MUP), neomycin (NEO), phenyl mercuric acetate (PMA), rifampicin, spectinomycin (SPT), streptomycin (STR), sulphonamide, tetracycline (TET), tobramycin (TOB), trimethoprim and vancomycin.

[d] Antimicrobial resistance and virulence genes were detected by DNA microarray profiling (Alere Technologies GmbH, Jena, Germany).

[e] Isolates M14/0965 and M15/0223 were recovered from the same patient on two separate occasions at hospital H1.

[f] Isolates M15/0540 and M15/0541, which were recovered from the same patient on two separate occasions, exhibited phenotypic susceptibility and resistance to mupirocin, respectively, despite mutual carriage of the mupirocin resistance-encoding gene, *mupA* (*ileS2*). This was due to an adenine base insertion after nucleotide position 283 in the *mupA* gene of M15/0540, which resulted in a downstream frameshift mutation and premature stop codon.

[g] Isolates M14/0148 and M15/0637 were recovered from the same patient on two separate occasions, at hospital H1 (M14/0148) and in the community by the patient's GP, (M15/0637).

[h] Patients had recent admission history to hospital H1.

[i] Patient presented to a GP but was a healthcare worker in H1.

Abbreviations: BSI, bloodstream infection; CA, community-associated; ED, emergency department; GP, general medical practitioner; H, hospital; HA, healthcare-associated; HCW, healthcare worker; LTCF, long-term care facility; N/A, not applicable; NH, nursing home; OHD, occupational health department; OPD, outpatient department; SSTI, skin and soft tissue infection; ST, sequence type.
